# Quantification and Analysis of the Irreversible Flow Loss in a Linear Compressor Cascade

**DOI:** 10.3390/e20070486

**Published:** 2018-06-22

**Authors:** Zhiyuan Li, Juan Du, Xavier Ottavy, Hongwu Zhang

**Affiliations:** 1 Department of Physics, University of Chinese Academy of Sciences, Beijing 100049, China; 2 Institute of Engineering Thermphysics, Chinese Academy of Sciences, Beijing 100190, China; 3 Laboratoire de Mécanique des Fluides et d’Acoustique, Ecole Centrale de Lyon, 69130 Ecully, France

**Keywords:** irreversible flow loss, entropy, cascade, computational fluid dynamics

## Abstract

A local loss model and an integral loss model are proposed to study the irreversible flow loss mechanism in a linear compressor cascade. The detached eddy simulation model based on the Menter shear stress transport turbulence model (SSTDES) was used to perform the high-fidelity simulations. The flow losses in the cascade with an incidence angle of 2°, 4° and 7° were analyzed. The contours of local loss coefficient can be explained well by the three-dimensional flow structures. The trend of flow loss varying with incidence angle predicted by integral loss is the same as that calculated by total pressure loss coefficient. The integral loss model was used to evaluate the irreversible loss generated in different regions and its varying trend with the flow condition. It as found that the boundary layer shear losses generated near the endwall, the pressure surface and the suction surface are almost identical for the three incidence angles. The secondary flow loss in the wake-flow and blade-passage regions changes dramatically with the flow condition due to the occurrence of corner stall. For this cascade, the secondary flow loss accounts for 26.1%, 48.3% and 64.3% of the total loss for the flow when the incidence angles are 2°, 4° and 7°, respectively. Lastly, the underlying reason for the variation of the secondary flow loss with the incidence angle is explained using the *L_c_* iso-surface method.

## 1. Introduction

For decades, the total pressure loss coefficient and entropy loss coefficient have played an important role in the study of flow losses in turbomachines. Based on the adiabatic hypothesis, these parameters have been successfully used to evaluate the global loss generated in the blade passage and to locate the low-energy fluid accumulation zones [1,2]. However, these two quantities do not allow quantification of the ratio of different loss sources in the flow field or analysis of the distribution of the local flow loss. To understand the origins of flow losses, a series of local loss models have been presented starting in the 1980s. These local loss models have been constructed based on the concept of entropy generation rate, enabling the intensity of irreversible loss in the internal flow to be evaluated according to the second law of thermodynamics [3]. The first local loss model, proposed by Moore [4], is based on the Reynolds-averaged Navier–Stokes (RANS) method. It assumes that the turbulent fluctuations of the heat flux and viscous dissipation in the positive definite entropy equation can be modeled by incorporating turbulent conductivity and turbulent viscosity to the molecular conductivity and viscosity, respectively. The loss mechanisms of the hub and shroud sealing flows [5] and tip shroud cavity flows [6] in turbines have been studied using this method. However, the Moore model assumes that the production of turbulent kinetic energy is equal to the turbulent dissipation rate. According to the energy cascade theory, this hypothesis is reasonable only in the inertial subrange of the flow field. Therefore, the applicability of the Moore model for the loss mechanism analysis in practical flows is limited. However, the construction theory of the Moore model can provide insight when developing more comprehensive methods. To overcome the limitations of the Moore model, Kramer-Bevan [7] and Herwig [8] proposed a new model to solve the entropy generation rate equation for the RANS simulation. The turbulent dissipation rate is directly calculated based on the turbulent model in this new model. Takakura [9] used the Kramer-Bevan model to study the loss mechanism in the tip region flow of a high-pressure turbine.

These studies emphasize the value of the local loss model for analyzing the irreversible loss mechanism of turbomachine flows. All of these local loss models have been based on the RANS method. However, many studies have shown that the RANS method cannot accurately predict the large separating flow, which is generally the most important factor associated with flow loss. Therefore, a local loss model constructed based on more precise CFD results has become increasingly necessary for quantifying the local loss in separating flows. Relative to the RANS method, direct numerical simulation (DNS), large-eddy simulation (LES) and hybrid RANS/LES methods are more accurate. Due to the limits of computational power, the hybrid RANS/LES method is the only one that is compatible with practical engineering applications in the coming decades [10]. Therefore, the motivation of this work is to build a local loss model based on the hybrid RANS/LES method. With the local loss model, it is possible to quantitatively predict the local flow loss generated in the flow fields and demonstrate the cause-and-effect relationship between the loss intensity and three-dimensional (3D) complex flow structures. 

In the present study, the equations of local and integral loss models based on the SSTDES turbulence model were derived and applied to loss analysis in a linear compressor cascade. After the introduction of the numerical configuration, the accuracy of the computational results and the reliability of the local and integral loss models were validated. The loss mechanism with various incidence angles in the compressor cascade was investigated. The quantitative analysis of loss sources was conducted by integrating the local loss coefficient in the entire computational domains. The mechanism for the major loss generated in the blade passage was clarified by examining the local 3D flow structure. Finally, the main results were concluded and discussed.

## 2. Local and Integral Loss Models Based on a Hybrid RANS/LES Model

In this section, the derivation of local and integral loss models based on the hybrid RANS/LES is presented. Since the SSTDES model is used to solve turbulence flow, as an example, the unknown turbulent quantities is defined according to the theory of the SSTDES model.

### 2.1. Entropy Generation Rate

As mediated by the second law of thermodynamics, entropy changes in a fluid can be represented by the entropy transport equation [11]. This equation is defined as

(1) $$\frac{\partial \left(\rho s\right)}{\partial t}+\frac{\partial \left(\rho u_{i}s\right)}{\partial x_{i}}=\frac{\partial }{\partial x_{i}}\left(\frac{k}{T}\frac{\partial T}{\partial x_{i}}\right)+\frac{k}{T^{2}}\frac{\partial T}{\partial x_{i}}\frac{\partial T}{\partial x_{i}}+\frac{2\mu s_{ij}s_{ij}}{T}$$

where *s* is the entropy, ρ is the density, $u_{i}$ is the velocity, k is the thermal conductivity, *T* is the temperature μ is the dynamic viscosity, and $s_{ij}\;$ is the strain-rate tensor. The last two terms of Equation (1) are always nonnegative; they represent entropy generation due to internal irreversibility. The first term, $\frac{k}{T^{2}}\frac{\partial T}{\partial x_{i}}\frac{\partial T}{\partial x_{i}}$, is the entropy generation rate due to heat transfer. The second term, $\frac{2\mu s_{ij}s_{ij}}{T}$, is the entropy generation rate due to viscous shear. These terms together describe the local loss intensity in flows, which is defined as

(2) $${\dot{S}}^{\prime \prime \prime }={\dot{S}}_{therm}^{\prime \prime \prime }+{\dot{S}}_{visc}^{\prime \prime \prime }==\frac{k}{T^{2}}\frac{\partial T}{\partial x_{i}}\frac{\partial T}{\partial x_{i}}+\frac{2\mu s_{ij}s_{ij}}{T}$$

For the flow fields calculated by DNS, Equation (2) can be calculated through post-processing as all the variables in this equation are solved explicitly. However, for the flow fields simulated using RANS or LES, only the Reynolds-averaged variables or subgrid filtered variables are obtained from the simulation results. Equation (2) cannot be used directly to calculate the entropy generation rate. According to the research of Kramer-Bevan [7] and Herwig [8], each term in Equation (2) can be split into two terms for use with the RANS method. One term is associated with mean quantities, and the other term is associated with fluctuating quantities as follows:

(3) $${\dot{S}}_{therm}^{\prime \prime \prime }=\frac{k}{{\bar{T}}^{2}}\frac{\partial \bar{T}}{\partial x_{i}}\frac{\partial \bar{T}}{\partial x_{i}}+\frac{k}{{\bar{T}}^{2}}\bar{\frac{\partial T^{\prime }}{\partial x_{i}}\frac{\partial T^{\prime }}{\partial x_{i}}}$$

(4) $${\dot{S}}_{visc}^{\prime \prime \prime }=\frac{2\mu {\bar{s}}_{ij}{\bar{s}}_{ij}}{\bar{T}}+\frac{2\mu \bar{s_{ij}^{\prime }s_{ij}^{\prime }}}{\bar{T}}$$

where ${\bar{s}}_{ij}$ and $s_{ij}^{\prime }$ are the mean and fluctuating rates of strain, respectively, and $\bar{T}$ and $T^{\prime }$ are the mean temperature and fluctuating temperature, respectively. In this study, the hybrid RANS/LES model was used as the turbulence model. The model consists of two computational regions: the RANS and LES domains. For this type of model, a subgrid filter should also be constructed. Equations (3) and (4) can be used regardless of the local averaging procedure. The terms with mean quantities in Equation (3) and (4) can be calculated by time-averaged quantities in the RANS calculation region and by filtered quantities in the LES calculation region. However, the terms with fluctuating quantities of Equation (3) and (4) cannot be explicitly solved using the hybrid RANS/LES method. According to the study of Herwig [8], the fluctuating variables can be described with the mean quantities and the turbulence characteristic parameters. In this way, the entropy generation rate in the temperature field ${\dot{S}}_{therm}^{\prime \prime \prime }\;$ and in the shear flow fields ${\dot{S}}_{visc}^{\prime \prime \prime }$ can be derived:

(5) $${\dot{S}}_{therm}^{\prime \prime \prime }=\frac{k}{{\bar{T}}^{2}}\frac{\partial \bar{T}}{\partial x_{i}}\frac{\partial \bar{T}}{\partial x_{i}}+\frac{\alpha_{t}}{\alpha }\frac{k}{{\bar{T}}^{2}}\frac{\partial \bar{T}}{\partial x_{i}}\frac{\partial \bar{T}}{\partial x_{i}}$$

(6) $${\dot{S}}_{visc}^{\prime \prime \prime }=\frac{2\mu {\bar{s}}_{ij}{\bar{s}}_{ij}}{\bar{T}}+\frac{\varepsilon \rho }{\bar{T}}$$

where $\alpha_{t}$ is the turbulent thermal diffusivity, α is the thermal diffusivity, ε is the turbulent dissipation rate, and ρ is the density. All variables in Equations (5) and (6) are explicitly solved except for ε, which represents the dissipation term of the turbulence kinetic transport equation in the RANS mode and the dissipation term of the subgrid scale kinetic energy transport equation in the LES mode. For the SSTDES model, which was used in this study, ε can be calculated as follows:

(7) $$\varepsilon =\beta^{*}k\omega F_{DES}\;\mathrm{with}\;F_{DES}=max\left(\frac{L_{t}}{C_{DES}\Delta }\left(1-F_{SST}\right),1\right),L_{t}=\frac{\sqrt{k}}{\beta^{*}\omega }$$

where $C_{DES}$ and $\beta^{*}$ are constants equaling 0.61 and 0.09, respectively, Δ is the filter-width, and FSST is one of the blending functions of the SST model. Details can be found in Reference [12].

### 2.2. Local Loss Model

As the shear stress in the viscous sublayer (the region where y^+^ < 5) is much larger than that in the main flow, the difference between the maximum and minimum entropy generation rate is considerable. Absolute entropy generation rate cannot provide the detailed flow information for the loss analysis. To solve this issue, the entropy generation rate due to viscous shear inside the viscous sublayer at the inlet was used to normalize the entropy generation rate over the entire flow fields. There are two advantages of using this value as the reference value: (1) its value is sufficiently large to evaluate the magnitude of the entropy generation rate; and (2) the value can be calculated using basic turbulent theory.

Since the turbulent fluctuation inside the viscous sublayer is negligibly small, the terms due to fluctuation in Equation (6) can be neglected. Thus, the entropy generation rate density in the viscous sublayer can be simplified as

(8) $${\dot{S}}_{visc,ref}^{\prime \prime \prime }=\frac{2\mu {\bar{s}}_{ij}{\bar{s}}_{ij}}{\bar{T}}+\frac{\varepsilon \rho }{\bar{T}}=\frac{2\mu {\bar{s}}_{ij}{\bar{s}}_{ij}}{\bar{T}}=\frac{1}{\bar{T}}\mu {\left(\frac{\partial \bar{U_{x}}}{\partial y}\right)}^{2}$$

where $\bar{U_{x}}$ is the streamwise velocity and *y* is the direction normal to the wall. ${\dot{S}}_{visc,ref}^{\prime \prime \prime }$ can be calculated once the gradient of the streamwise velocity is known. The skin-friction coefficient (*C_f_*) was used to evaluate this gradient. According to [13], *C_f_* is defined as

(9) $$C_{f}=\frac{2\tau_{w}}{\left(\rho U_{0}^{2}\right)}\;\mathrm{with}\;\tau_{w}=\mu {\left(\frac{\partial \bar{U_{x}}}{\partial y}\right)}_{y=0}$$

where *U*_0_ is the velocity of the main flow. Furthermore, according to the Blasius friction law [14], *C_f_* of the internal flow with 1 × 10^4^ < Re < 5 × 10^6^ can be calculated as

(10) $$C_{f}=2{\left(2.236\mathrm{lnRe}-4.639\right)}^{-2}$$

where Re is the Reynolds number at the inlet. According to the relationship at the wall [13], the velocity gradient is a constant inside the viscous sublayer. Thus,

(11) $${\left(\frac{\partial \bar{U_{x}}}{\partial y}\right)}_{y^{+}<5}={\left(\frac{\partial \bar{U_{x}}}{\partial y}\right)}_{y=0}$$

where *x* is the streamwise direction and *y* is the direction normal to the wall. Substituting Equations (9)–(11) into Equation (8), the referenced entropy generation rate can be calculated as follows:

(12) $${\dot{S}}_{visc,ref}^{\prime \prime \prime }=\frac{\bar{\rho^{2}U_{0}^{4}C_{f}^{2}}}{4\mu \bar{T}}$$

In this way, Equation (12) can be used as a reference parameter to define a local loss model:

(13) $$L_{c}=\frac{{\dot{S}}^{\prime \prime \prime }}{{\dot{S}}_{visc,ref}^{\prime \prime \prime }}=\frac{4\mu \bar{T}{\dot{S}}^{\prime \prime \prime }}{\rho^{2}U_{0}^{4}C_{f}^{2}}$$

where ${\dot{S}}^{\prime \prime \prime }$ is obtained by combining Equations (5) and (6). In this study, *L_c_* was used to locate regions of high flow loss in the flow fields.

### 2.3. Integral Loss Model

As mediated by the second law of thermodynamics, the irreversible loss generated in domain *V* can be calculated as

(14) $$Loss=\int_{V}{\dot{S}}^{\prime \prime \prime }T_{0}dV=\overset{n}{\underset{0}{\sum }}{\dot{S}}_{i}^{\prime \prime \prime }T_{0}V_{i}$$

where *V* represents a user-defined flow domain, ${\dot{S}}^{\prime \prime \prime }$ is obtained by combining Equations (5) and (6), and *T*_0_ is the environment temperature. For the numerical simulation, the integral computation can be solved by a summation of the value in every mesh cell, as shown by the leftmost part of Equation (14). *i* represents an arbitrary mesh cell and *n* is the total number of mesh cell. The units of the integral loss are *W*. This parameter can be used to quantify the flow loss in arbitrary flow regions when the flow fields are solved.

## 3. The Studied Cascade and Numerical Configuration

### 3.1. The Studied Cascade

**Figure 1:**
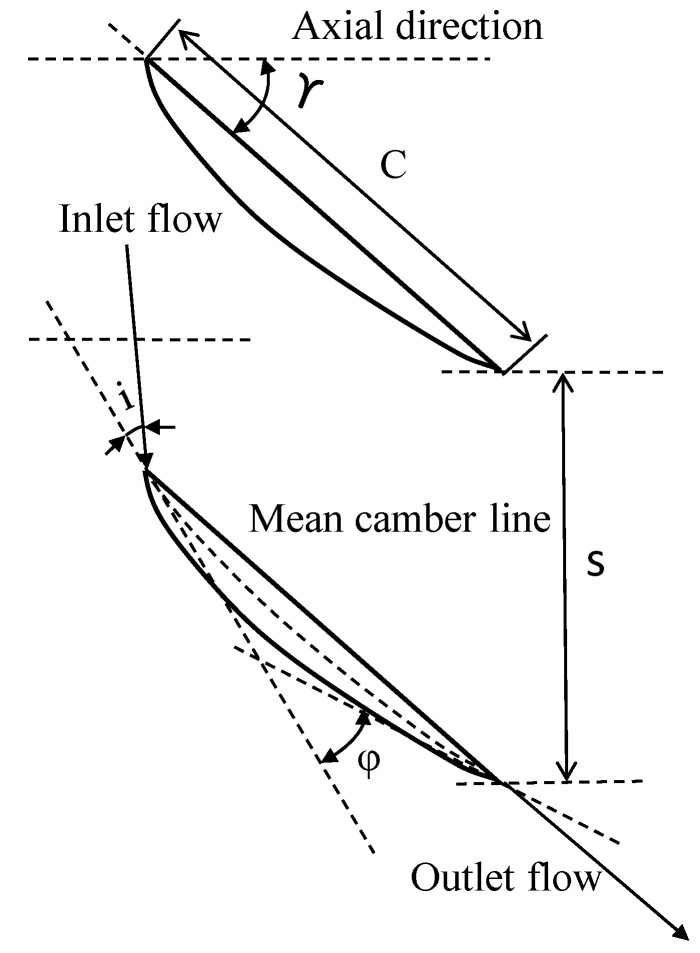
Notation for cascade.

The geometric parameters of the studied cascade are shown in Table 1. The detailed experiments have been performed by Laboratory of Fluid Transfer and Acoustics (LMFA) at Ecole Centrale Lyon such as in studies by Ma et al. [15] and Zambonini and Ottavy [16,17]. The cascade is a linear compressor cascade with a 150 mm chord and 370 m blade span. The flow in the cascade can be regarded as an incompressible flow with a free-stream velocity of 40 m/s at the inlet. The turbulence intensity is approximately 0.8% in the free-stream. The incidence angles of 2°, 4° and 7° were chosen for the present study. A significant corner separation is observed when the incidence angle is larger than 4°. The notation of cascade is presented in Figure 1, where *C* is chord; *γ* is stagger angle; *ϕ* is camber line; *i* is incidence angle; and *s* is pitch spacing.

### 3.2. Numerical Method

Open-source CFD software, OpenFOAM 2.2.2, was used to conduct the numerical simulations. The pimpleFoam code was selected as the computational solver. The reliability of the solver was tested previously [18,19]. The SSTDES model derived from the SST *k*-ω model was used to solve the Navier–Stokes equations. The LES method was adopted in the central region, while the RANS method was used to treat the near-wall region. A shielding function was used to distinguish the computational zone of RANS and LES. 

The zero gradient pressure boundary condition and the fixed velocity boundary condition were applied at the inlet. The velocity profile at the inlet is the same as that of the experimental data [15]. The relative static pressure at the outlet is zero. The outside normal velocity at the outlet is zero. The inlet turbulence kinetic energy and turbulence specific dissipation rate are 1.6224 m^2^s^−2^ and 3134 s^−1^, respectively. No-slip boundary condition was used on the wall.

**Figure 2:**
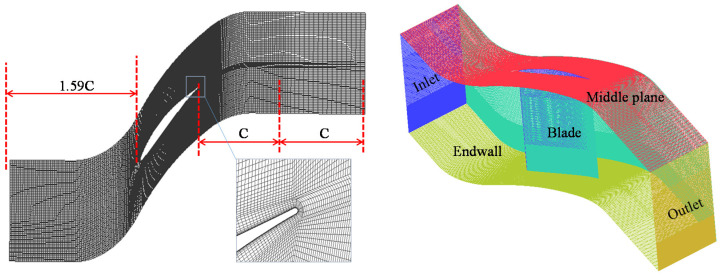
The computational model with the fine mesh.

The Gauss linear upwind stabilized transport unlimited Grad(U) scheme was used to interpolate the convective term. The interpolate schemes of the dissipation term are second-order central scheme. The Courant–Friedrichs–Lewy (CFL) number is less than 1. The unsteady computational physical time is 40 times of the time the fluid flow through the computational domain. To guarantee sufficient resolution in the boundary layer, y^+^ of the first near-wall layer of the computational mesh is less than 1. The mesh in the region close to the cascade trailing edge was further refined to capture the corner separation flow details. To reduce the reflective behavior of the outlet boundary, the grids in the outlet region were stretched to introduce artificial dissipation. To reduce the computational cost, only half of the blade span was used to conduct this simulation using the symmetric boundary condition. The lengths of the inlet and outlet regions are 1.59C and 2C, respectively, where C is the chord length of 150 mm. The grid number is 5.8 million, which has been proven to be mesh independent (see [20] for details). The mesh is shown in Figure 2.

## 4. Validation of the Simulation Results

In this section, the accuracy of the SSTDES method is evaluated by comparing the numerical results at the incidence angle of 4° with the experimental data. In addition, the reliability of the local loss coefficient and the integral loss model are verified.

### 4.1. Time-Averaged Static Pressure Coefficient on the Blade

**Figure 3:**
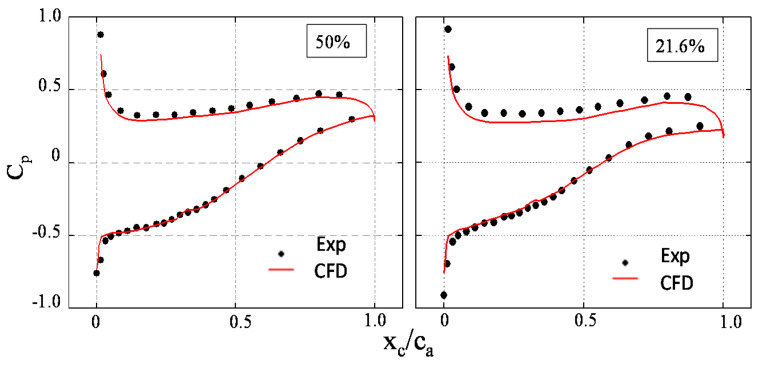
The static pressure coefficient (*C_p_*) on the blade at 50% and 21.6% span.

Figure 3 depicts the time-averaged static pressure coefficient (*C_p_*) on the blade of the experiments and CFD results at 50% and 21.6% blade span. The *C_p_* on the blade reflects the blade load distribution, defined as

(15) $$C_{p}=\frac{p-p_{\infty }}{p_{t\infty }-p_{\infty }}$$

where *p* is the local static pressure and $p_{\infty }$ and $p_{t\infty }$ are the inlet static pressure and inlet total pressure at the middle span, respectively.

**Figure 4:**
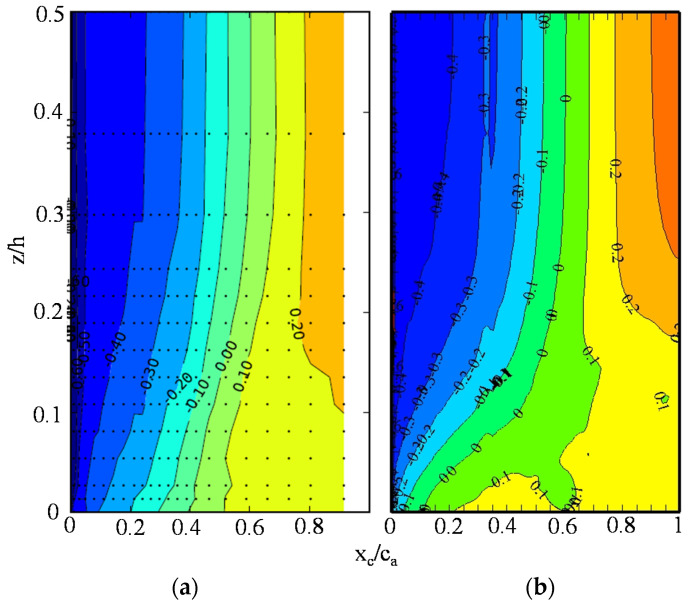
Comparison diagrams between the measured and calculated static pressure coefficient on the suction surface: (**a**) Exp (Ma [21]); and (**b**) CFD.

In Figure 3, the abscissa is the dimensionless axial position. x_c_ is the axial direction of the cascade frame. c_a_ is the axial chord length. The position of x_c_/c_a_ = 0 and x_c_/c_a_ = 1 are for the blade leading edge and trailing edge, respectively. The ordinate is the time-averaged *C_p_*. The black points represent the experimental data, whereas the red line represents the data calculated by the SSTDES model. The *C_p_* distributions calculated by the SSTDES model are close to the experimental data, and the trend of the simulation results is consistent with the experiments. Figure 4 depicts the measured and calculated *C_p_* contours on the suction surface. It is shown that the calculated distribution of *C_p_* on the suction surface is close to the experimental data. In general, the SSTDES model can predict the *C_p_* distribution on the blade with acceptable accuracy.

### 4.2. Local Loss Coefficient and Integral Loss

The SSTDES results provide accurate predictions of time-averaged static pressure coefficient on the blade and the surface limiting streamline, as described above. In this section, the local loss model, defined by Equation (13), and the integral loss model, defined by Equation (14), are used to test the reliability of the computational data. First, the distribution of the local loss model behind the trailing edge at an incidence angle (i) of 2°, 4° and 7° is analyzed and related with the 3D flow structures in the corner region.

**Figure 5:**
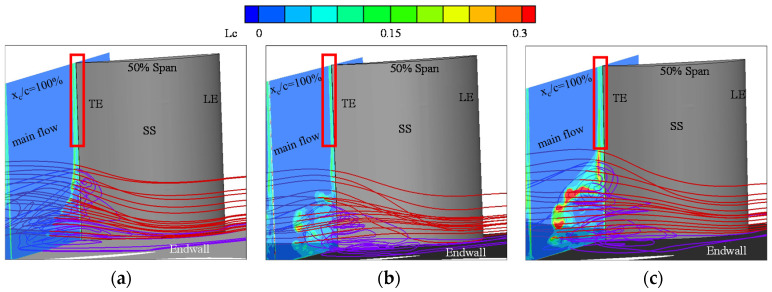
Distribution of the local loss coefficient with the 3D streamlines in the cascade passage: (**a**) i = 2°; (**b**) i = 4°; and (**c**) i = 7°.

Figure 5 presents the distribution of the local loss coefficient behind the trailing edge (x_c_/c_a_ = 1) with the 3D streamlines near the corner stall region. The local loss coefficient is defined by Equation (13). Figure 5a–c shows the results at the incidence angles of 2°, 4° and 7°, respectively. According to the study of Lei et al. [22], the cross-flow from the pressure side to suction side is due to the overturning of fluid close to the end-wall inside the blade passage, which transfers low-energy fluid to the corner region. The purple streamlines in each plot represent the low-energy flow in the corner region. The main flow structure in the corner region is a tornado vortex. The red streamlines represent the main flow outside the tornado vortex. The loss intensity is enhanced, and the domain of the high-loss-intensity region is increased with increasing incidence angle. For the main flow, the loss intensity is essentially negligible. Behind the trailing edge and far from the corner separation region (shown by the red rectangles), the shear interaction between the boundary layers of the blade suction surface and the blade pressure surface generates considerable loss. Another high-loss region at x_c_/c_a_ = 1 is the region outside the boundary of the tornado vortex. This phenomenon is caused by the mixing between the tornado vortex and the main flow due to the large difference in velocity between these two types of flows.

**Figure 6:**
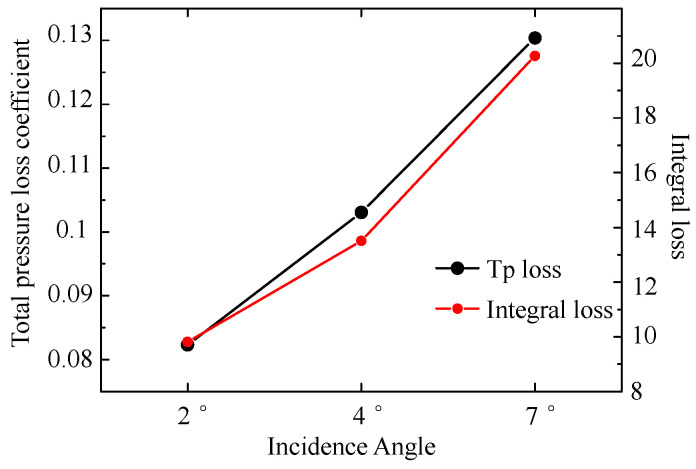
Total pressure loss coefficient and integral loss of the whole computational domain in 2°, 4° and 7° incidence angles.

The loss data at the three incidence angles are extracted from numerical results and plotted in terms of the total pressure loss coefficient and integral loss curves as a function of incidence angle in Figure 6. Both the total pressure coefficient and the integral loss can represent the flow loss in the whole computational domain. The definition of the total pressure coefficient is as:

(16) $$\omega_{c}=\frac{P_{t\infty }-p_{t}}{\frac{\rho U_{\infty }^{2}}{2}}$$

where, $P_{t\infty }$ represents the total pressure at the inlet; *P_t_* is the total pressure at a specific position; $U_{\infty }$ denotes the velocity at the inlet. The quantity of loss increases with increasing incidence angle, and the trend of integral loss calculated by Equation (14) based on the entropy generation rate matches well with the total pressure loss coefficient curve. Therefore, we conclude that the local and integral loss models can be used to analyze the flow loss mechanism. The reliability of the computational data was tested. Further experimental validation for the CFD results can be found in Reference [20]. 

## 5. Loss Mechanism Variation with Incidence Angle

The local loss variation with flow incidence angles in a cascade is studied in detail in this section. First, the loss sources at various regions in the entire computational domain are quantitatively evaluated by integrating the local loss coefficient. The variation trend of the fraction of each loss source with the incidence angle is also discussed. Then, the distribution of flow loss along the spanwise direction in the regions that are responsible for the two largest amounts of flow loss is analyzed. Finally, the iso-surface of local loss coefficient labelled with the axial velocity and with 3D streamlines is used to provide the interpretation for the loss mechanism at various incidence angles.

### 5.1. Quantitative Analysis of Loss Sources 

**Figure 7:**
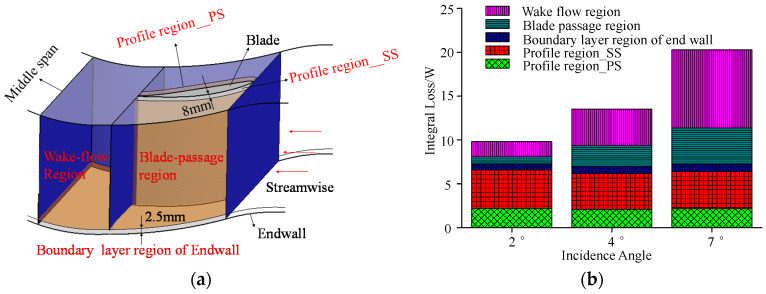
Integral region and proportion of five types of loss sources: (**a**) integral regions; and (**b**) proportion of various loss sources.

The computational domain from the blade leading edge to the 0.8 times of axial chord downstream the trailing edge, is divided into five integral regions: the endwall region, the profile region near the pressure surface (PS), the profile region near the suction surface (SS), the blade-passage region and the wake-flow region. The 3D schematic diagram of the five integral regions is shown in Figure 7a. To include the main the main viscous shear loss in the boundary layers, the thicknesses of the endwall region and the profile region are set to be 2.5 mm and 8 mm, respectively. The thickness values in these regions are identified by the *L_c_* contour in [20]. The wake-flow region is defined by 1 < x_c_/c_a_ < 1.8. The blade-passage region shown in Figure 7a is assumed to be dominated by the secondary flow loss in the cascade passage. Equation (14) is used to calculate the amount of loss generated in these regions. Figure 7b presents their proportions for flows at three incidence angles. 

For each loss region shown in Figure 7b, the loss intensity is not always enhanced when the incidence angle increases. The losses generated in the endwall region, the profile region of suction surface and the profile region of pressure surface, which can be classified as the shear loss in the boundary layer, are only minimally influenced by the incidence angle. The significant change of losses occurs in the wake-flow region and the blade passage. The loss generated in these two regions is traditionally regarded as the secondary flow loss. These two loss types are enhanced dramatically with the growth of the incidence angle. For the flow with a 2° incidence angle, the total intensity of the secondary flow loss is approximately 2.564 Watt. However, the intensity is approximately 13.034 Watt for the flow with a 7° incidence angle, which is almost five times the former value. The secondary flow loss accounts for 26.1%, 48.3% and 64.3% of the total loss for the flow with 2°, 4° and 7° incidence angles, respectively. The loss distribution in the wake-flow region and the blade passage is studied in detail in the following section.

### 5.2. Loss Generated in the Wake-Flow Region, and the Blade Passage 

**Figure 8:**
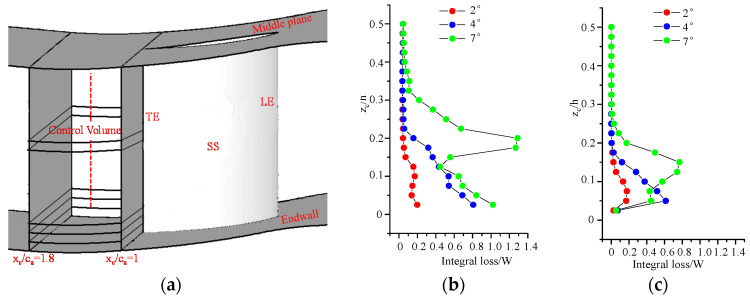
Integral loss along the dimensionless cascade span: (**a**) schematic of control volumes; (**b**) loss distribution in the wake-flow region; and (**c**) loss distribution in the cascade passage.

The curves in Figure 8b represent the distribution of the integral wake-flow loss along the blade spanwise direction of the wake-flow region. The integral wake-flow loss is calculated using Equation (14). A schematic of the control volumes in the wake-flow region is shown in Figure 8a. The domain of the wake-flow region is divided equally along the blade span, generating a total of 20 control volumes. The control volumes cover one pitch of the cascade passage in the circumferential direction and extend from the blade-trailing edge to 0.8c_a_ downstream of the blade-trailing plane in the axial direction. The regions of high loss intensity of the flows with incidence angles of 2°, 4° and 7° are located in the regions of 0 < z_c_/h < 0.15, 0 < z_c_/h < 0.225 and 0 < z_c_/h < 0.325, respectively. In other words, the high-loss-generating region is expanded. For incidence angles of 2° to 4°, the distance to the endwall is inversely correlated with the wake-flow loss intensity. In the spanwise region of z_c_/h < 0.125, the loss intensity in the wake-flow region of the flows with incidence angles of 4° and 7° are similar. However, when z_c_/h > 0.125, the loss intensity of the flow with an incidence angle of 7° is much stronger than the flow with an incidence angle of 4°. With the same post-processing method as that used in Figure 8b, Figure 8c presents the distribution of the integral secondary flow loss along the blade spanwise direction in the blade passage. The secondary-flow loss intensity in the cascade passage is less than that of the wake flow shown in Figure 8b. In the cascade passage, the high-loss-intensity region of the three incidence angles are 0 < z_c_/h < 0.15, 0 < z_c_/h < 0.15 and 0 < z_c_/h < 0.25. Different from the trend of the loss generated in the wake-flow region, the loss intensities in the control volume of the blade passage close to the endwall are almost the same for the three incidence angles. Figure 4 and Figure 7c indicate that the high-loss regions correspond to the corner separation region. It is thus concluded that the high flow loss generated in the blade passage is caused primarily by the corner separation flow.

### 5.3. Analysis of 3D Loss Distribution 

**Figure 9:**
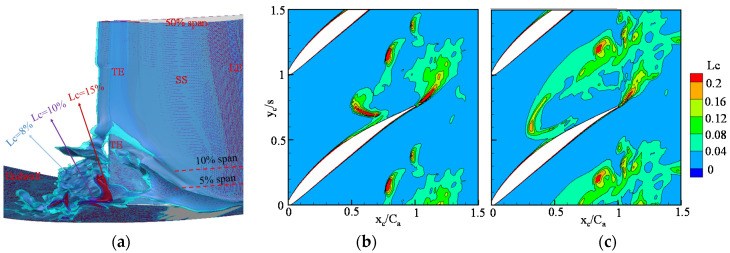
*L_c_* distribution of the flow with an incidence angle of 4°: (**a**) iso-surface of *L_c_* = 8%, 10% and 15%; (**b**) *L_c_* distribution at z/h = 0.1; and (**c**) *L_c_* distribution at z/h = 0.05.

Iso-surfaces are a useful way to show the 3D distribution of a quantity in a flow field if the distribution of the quantity is continuous. The Q criterion [23] is a popular tool for identifying the distribution of vortical structures according to LES and DNS results. In this way, if the local loss coefficient *L_c_* is continuous, researchers can also use the iso-surface method to show the local loss distribution in a flow field intuitively. Figure 9a presents the iso-surfaces of *L_c_* = 8%, 10% and 15% of the flow with an incidence angle of 4°. The iso-surfaces of *L_c_* = 8%, 10% and 15% are shown in blue, purple and red, respectively. The iso-surface of *L_c_* = 15% is contained in the iso-surface of *L_c_* = 10%, and the iso-surface of *L_c_* = 10% is contained in the iso-surface of *L_c_* = 8%. Figure 9b,c present the *L_c_* contours at z/h = 0.1 and the *L_c_* distribution at z/h = 0.05, respectively. The *L_c_* distribution in these two positions is continuous. The regions with large *L_c_* are contained in the regions with small *L_c_*. We therefore conclude that *L_c_* in an iso-surface is larger than that used to make the iso-surface. The iso-surface method can be used to evaluate the strength of the loss intensity

**Figure 10:**
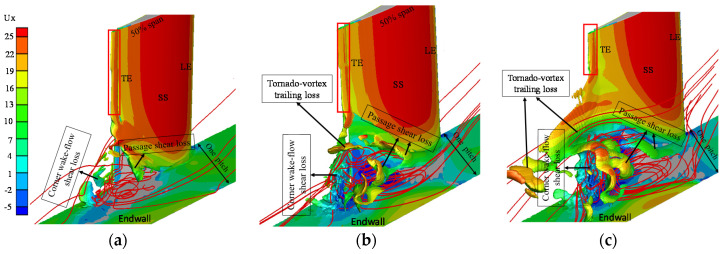
3D loss distributions of the flows with 2°, 4° and 7° incidence angles (*L_c_* = 0.1 with axial velocity (*U_x_*) rendered): (**a**) i = 2°; (**b**) i = 4°; and (**c**) i = 7°.

Figure 10a–c presents the loss distributions with the iso-surface of *L_c_* = 0.1 of the flow at the incidence angles of 2°, 4° and 7°, respectively. The iso-surface of *L_c_* = 0.1 is chosen because this value can depict the main features of local loss distribution, as shown in Figure 5 and Figure 9. The contours of axial velocity (*U_x_*) are shown together with the iso-surface. The streamlines are used to show the structures of the corner separation flows. The high-loss region is associated primarily with the corner separation flow regardless of the incidence angle. The domain of the high-loss region increases sharply with the growth of the incidence angle. When the wake flow is away from the corner region (as shown by the red rectangles), the high-loss region shrinks in the domain close to the trailing edge for the three types of flow. In the corner region, three regions exist where the loss intensity changes sharply with the growth of the incidence angle. One is the interface region between the main flow and the outside of the corner separation flow, here referred to as passage shear loss. The other one is the mixing region between the boundary layer flow of the pressure side and the corner separation flow, here termed the corner wake-flow shear loss. These two high-loss regions exist at all three incidence angles. For the flow at incidence angles of 4° and 7°, another high-loss region exists at the top of the corner separation flow where the tornado vortex exits the cascade passage. As shown in Figure 5 and elsewhere [24], the main flow structure in this region is the tail of the corner tornado vortex; thus, this type of loss is termed the tornado-vortex trailing loss.

For the flow with a 2° incidence angle, the passage shear loss occupies only the region on the top of the corner separation flow. With increasing incidence angle, the passage shear loss on the side of the corner separation flow is enhanced, and the top is strengthened simultaneously. For the corner wake-flow shear loss, this type of loss accumulates at the end of the blade near the end wall and is strengthened with the growth of the incidence angle. This phenomenon can be explained as follows: the conditions of the flow coming from the pressure side and the corner separation flow are more inconsistent when the incidence angle is increased. Therefore, the large changes of the flow conditions in this region result in a large entropy generation rate. The tornado-vortex trailing loss changes more than the other two types of loss with the growth of the incidence angle. This type of loss is not present for the flow with a 2° incidence angle. However, the wake-flow region is occupied by a huge high-loss cluster for the flow with a 7° incidence angle. The underlying mechanism of high loss generated in the tornado-vortex trailing loss region at 7° incidence angle, as shown in Figure 10c, is analyzed in detail in the following. 

**Figure 11:**
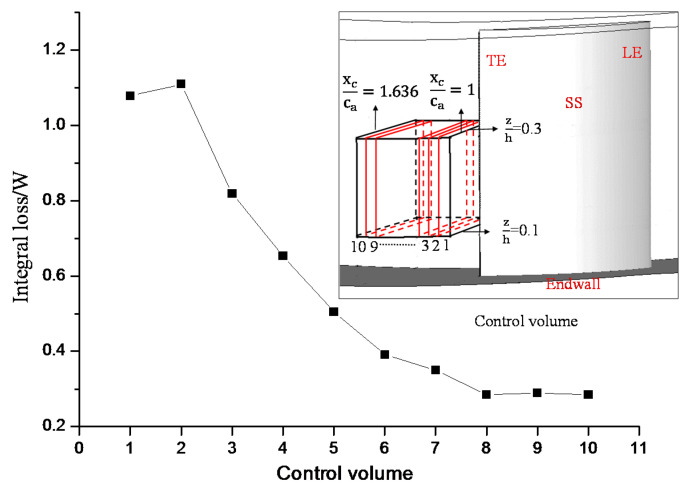
Integral loss along the streamwise direction in the tornado-vortex trailing loss region.

At the 7° incidence angle, the tornado-vortex trailing loss region is in the domain where $0.1<\frac{z}{h}<0.3$ and $1<\frac{x_{c}}{c_{a}}<1.636$ according to Figure 9a. To further study the evolution of loss intensity in this domain along the streamwise direction, the integral losses along the streamwise direction are presented in the Figure 11. In this region, 10 control volumes are equally divided along the streamwise direction. Equation (14) was used to calculate the integral loss. It is shown that closer to the blade trailing edge higher loss intensity is generated; most losses are generated in the first five control volumes where $1<\frac{x_{c}}{c_{a}}<1.32$. Therefore, the flow structures in the flow region where $1<\frac{x_{c}}{c_{a}}<1.32$ is studied to analyze the generation mechanism of high loss intensity in the tornado-vortex trailing loss region at the 7° incidence angle.

**Figure 12:**
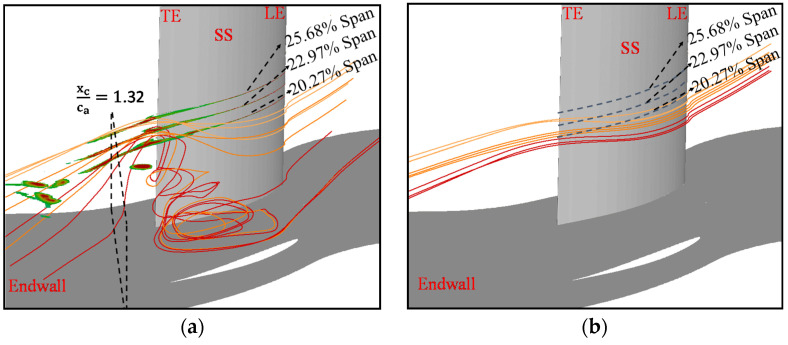
Flow structures of 4° and 7° incidence angle in the tornado-vortex trailing loss region: (**a**) i = 7°; and (**b**) i = 4°.

Figure 12a presents the main flow structures in the tornado-vortex trailing loss region at the 7° incidence angle. A black dotted box is drawn in Figure 12a to represent the position of $\frac{x_{c}}{c_{a}}=1.32$. The contours of *L_c_* larger than 0.1 at the 20.27%, 22.97% and 25.68% blade spans are plotted in Figure 12. They are used to depict the core of the tornado-vortex trailing loss. The streamlines with red, orange and yellow color are the ones released from the core of the high loss region at the 20.27%, 22.97% and 25.68% blade spans, respectively. The tornado-vortex structure is clearly depicted by the streamlines in the corner region near the suction side. It is shown that all the streamlines are deflected to the upstream when they approach to the tornado-vortex, and then are abruptly turned to the endwall. Meanwhile, the closer the streamlines are to the endwall, the larger the deflection angle is. In contrast, Figure 12b presents the streamlines released at the same positions as Figure 12a at the 4° incidence angle. One can see that there are no large deflection flows in the same region as the flow of 7° incidence angle. The difference can be explained like that: the corner separation of the 7° incidence angle is more serious than the 4° incidence angle, which results in the stronger effect of blockage to the main flow in the 7° incidence angle. In this way, the difference of pressure between the main flow and the flow downstream the corner separation region is larger at the 7° incidence angle, which makes the angle of deflection flow at the 7° incidence angle be larger than the one at the 4° incidence angle. According to the second law of thermodynamics, the larger the flow condition change is, the higher the irreversible losses are. Therefore, high losses are generated in the tornado-vortex trailing loss region at the 7° incidence angle.

## 6. Discussion

In this study, a local model and an integral loss model based on the SSTDES computational model were evaluated theoretically. Using a well-tested mesh, the accuracy of computational results was validated. The local and integral loss distribution varying with flow incidence angles were studied in detail. The main results are summarized as follows:

(1) The local and integral loss models, derived on a rigorous theoretical basis, can be used to quantify the local loss and analyze the loss mechanism once the simulation results calculated by the SSTDES model are independent of the computational mesh. The local loss-intensity distribution described by the local loss model can be explained well by the 3D flow structures. The trend of the total flow loss calculated by the integral loss model is the same as that exhibited by the total pressure loss coefficient with the incidence angle.
(2) The boundary layer shear loss is almost uninfluenced by the incidence angle. The secondary flow loss in the wake flow and blade-passage regions changes dramatically with the flow condition due to the occurrence of corner stall. For this cascade, the secondary flow loss accounts for 26.1%, 48.3% and 64.3% of the total loss for the flow at incidence angles of 2°, 4° and 7°, respectively.
(3) Insight can be derived using the iso-surface contours of *L_c_* to identify the 3D loss distribution. With increasing incidence angle, three types of secondary flow loss change dramatically. They are the passage shear loss, which is located at the interface region between the main flow and the outside of the corner separation flow; the corner wake-flow shear loss, which is located in the mixing region between the boundary layer flow of the pressure side and the corner separation flow; and the tornado-vortex trailing loss, which represents the loss generated at the top of corner separation flow where the main flow structure is the tail of the corner tornado vortex.

## Figures and Tables

**Table 1:** Geometric parameters of the cascade.

Parameter	Value	Parameter	Value
Chord (mm)	150	Blade span (mm)	370
Camber angle (°)	23.22	Aspect ratio	2.47
Stagger angle (°)	42.7	Design upstream flow angle (°)	54.31
Pitch spacing (mm)	134	Design downstream flow angle (°)	31.09
Solidity	1.12		

## Nomenclature

*C, c_a_*	blade chord, axial blade chord
*C_p_*	static pressure coefficient
*C_f_*	skin friction coefficient
*h*	blade span
*i*	incidence angle
*k*	turbulence kinetic energy
*L*	loss intensity
*L_c_*	local loss coefficient
*p*	pressure
*Re*	Reynolds number
*s*	pitch spacing
*s_ij_*	fluctuating strain-rate tensor
*S_ij_*	shear strain rate
$S_{visc}^{\prime \prime \prime }$	entropy generation of viscosity dissipation
$S_{therm}^{\prime \prime \prime }$	entropy generation of heat transfer
*U*	velocity
*x_c_, y_c_*	axial, longitudinal direction of cascade frame
*z_c_*	spanwise direction of cascade frame
*ρ*	density
*ϒ*	stagger angle
*ϕ*	Camber line
*υ*	kinematic viscosity
*μ*	dynamic viscosity
*ω*	specific dissipation rate
*τ_w_*	wall shear stress
*LE, TE*	leading edge, trailing edge
*PS, SS*	pressure surface, suction surface
